# TWEAK and TSLP in disc degeneration and spontaneous hernia resorption

**DOI:** 10.1002/jsp2.1068

**Published:** 2020-01-09

**Authors:** Tetsuro Ohba, Hirotaka Haro

**Affiliations:** ^1^ Department of Orthopaedic Surgery University of Yamanashi Yamanashi Japan

**Keywords:** disc degeneration, thymic stromal lymphopoietin, TNF‐like weak inducer of apoptosis

## Abstract

Spontaneous degeneration of an intervertebral disc is caused by inflammation that accompanies exposure of the avascular nucleus pulposus to circulation, triggering an autoimmune inflammatory reaction. Both intrinsic and extrinsic mechanisms of IVD regulation by various cytokines are involved in disc degeneration and spontaneous hernia resorption through inflammatory responses. The major goal of this narrative review was to assemble our past findings about the potential role of cytokines in disc diseases and to clarify directions for future research. A member of the tumor necrosis factor‐α (TNF‐α) superfamily, TNF‐like weak inducer of apoptosis (TWEAK) is constitutively expressed in the intervertebral disc, and induces a chronic, but relatively weak inflammatory response, thereby suppressing the formation of cartilage matrix and inducing production of matrix metalloproteinases (MMPs). Previously we indicated that TWEAK is involved in intervertebral disc degeneration by inhibiting the production of cartilage matrix in the intervertebral disc, and inducing the further expression of MMP‐3. Thymic stromal lymphopoietin (TSLP) is expressed primarily by epithelial cells, and induces inflammation at the time of tolerance failure in allergic disease. We found TSLP induced migration of immunocompetent cells to the disc in intervertebral disc disease by promoting the production of monocyte chemoattractant protein‐1 (MCP‐1) and macrophage inflammatory protein‐1 alpha (MIP‐1α) by the intervertebral disc and these cells may be involved in the resorption of herniated disc tissue. Considering the pivotal role of TWEAK and TSLP we review our current understanding of these factors and their involvement in disc degeneration.

## 1 INTRODUCTION

With the aging of society, the number of people requiring treatment for low back pain is increasing.^1^ It is an urgent task to reduce musculoskeletal disorders caused by low back pain, avoid the requirement for assistance with daily living, and extend the healthy life span of affected individuals. In disc herniation, a degenerated disc protrudes into the spinal canal and can cause back pain or leg pain by pressing upon a nerve root or the cauda equina. Disc degeneration has been described as an etiology of low back pain.^2^, ^3^ Inflammation associated with disc degeneration and sensory nerve penetration into the disc may also contribute to back pain, and therefore the disc is considered to be a primary target for treatment.^2^, ^3^

By elucidating the mechanisms of disc degeneration, a new approach to low back pain may become possible, and if so, the benefits to society will be considerable. However, anti‐ or cytokine therapy for disc degeneration disease is not yet established or applied clinically. Therefore, a more detailed understanding of the role of cytokines in disc degeneration would be valuable. In intervertebral disc herniation, spontaneous retraction of the hernia mass has been confirmed,^4^, ^5^ and is linked to immunocompetent cells such as macrophages that infiltrate the hernia mass and are accompanied by local inflammation.^4^, ^5^, ^6^

Major inflammatory cytokines such as tumor necrosis factor‐α (TNF‐α) and interleukin‐1β (IL‐1β) may contribute not only to the natural regression of hernia masses,^7^, ^8^ but also to the mechanism of intervertebral disc degeneration. However, how best to target these factors for therapeutic strategies remains to be determined. Here we review the involvement of cytokines in inflammation and degeneration mechanisms in the intervertebral disc that we have identified so far. The major goal of this narrative review is to assemble our past findings and to clarify directions for future research.

In previous reports, we described our established “mouse disc tissue culture,” that enabled us to extract discs from the caudal vertebrae of the mouse under a microscope, and culture and analyze the tissue under various stimulation by cytokines.^5^ DNA and proteins could be extracted from the cultured intervertebral disc tissue, and the levels of expression of cytokines, aggrecan, and type 2 collagen were quantified. The intervertebral discs were also evaluated histologically by safranin O and immunostaining. Later, we reapplied this strategy to herniated human IVD samples resected surgically, and these were examined histologically.^9^

**Figure 1:**
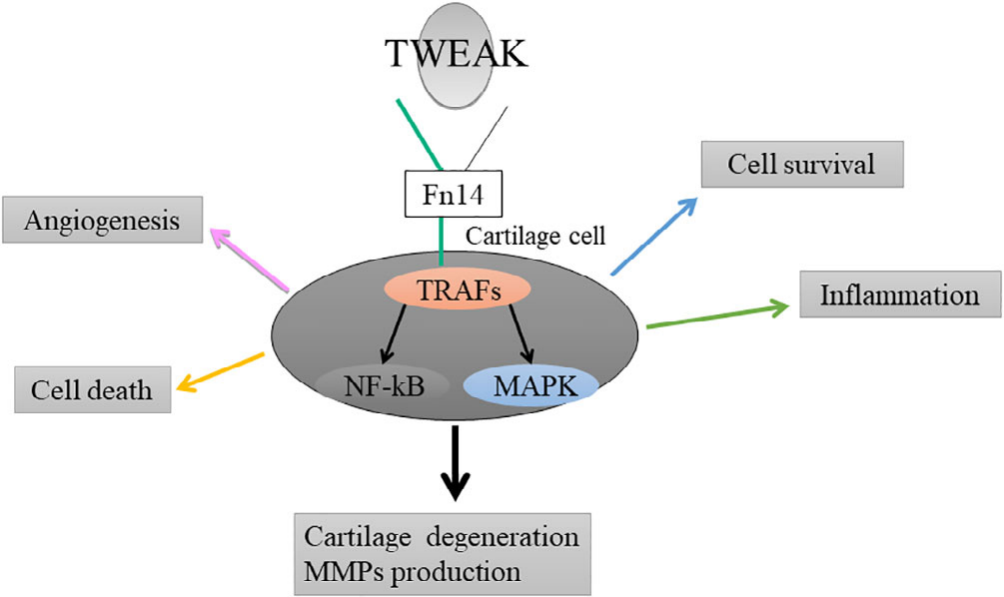
The effect of multifunctional TWEAK and Fn14 signaling on cartilage reported in previous articles is shown schematically. TWEAK, TNF‐like weak inducer of apoptosis; TRAFs, TNF‐receptor associated factors; NF‐κB, nuclear factor‐kappa B; MAPK, mitogen‐activated protein kinase

We found that disc degeneration induces TNF‐like weak inducer of apoptosis (TWEAK) and Fn14 signaling.^10^ The effect of multifunctional TWEAK and Fn14 signaling on cartilage is shown schematically in Figure 1. TWEAK is a member of the TNF‐α superfamily of cytokines discovered in 1997 and is primarily expressed as a type II transmembrane protein. TWEAK binds to Fn14 (CD266), a receptor whose expression has been confirmed in many cells.^11^ TWEAK mainly controls cell survival and apoptosis through the signal activity of MAP and NF‐κB, and whose activation induces inflammation.^11^ TWEAK has various functions such as stimulating proliferation, migration, angiogenesis, differentiation and the expression of proinflammatory cytokines.^11^, ^12^, ^13^ Among them, TWEAK is expressed in articular cartilage in a mouse model of arthritis and may be involved in cartilage degeneration.^11^ It is also interesting to note that TWEAK induces matrix metalloproteinases (MMPs) in several cell types.^13^

### 1.1 Roles for TWEAK in disc degeneration suggested by our research

**Figure 2:**
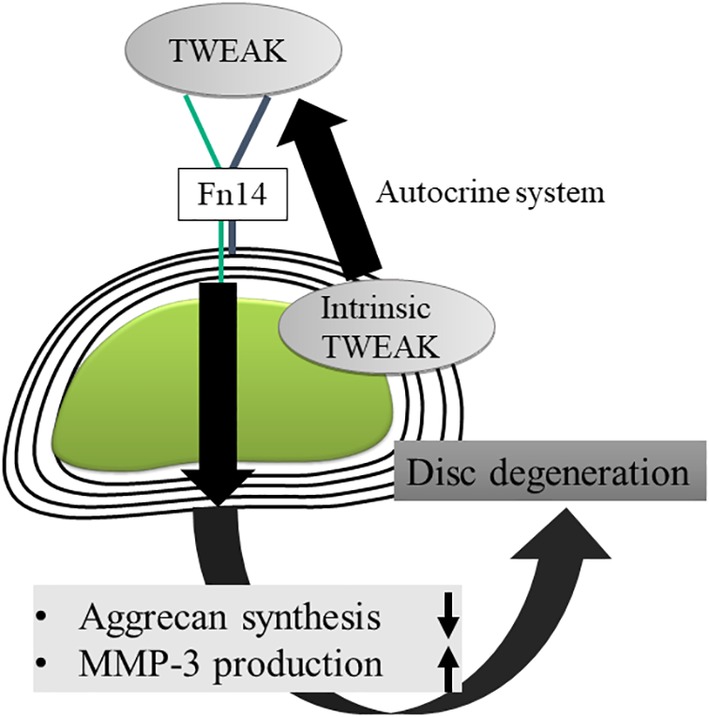
Schematic overview of a proposed mechanism of disc degeneration by TWEAK and Fn14 signaling based on our previous studies. TWEAK is involved in degeneration of the intervertebral disc by promoting degradation of the cartilage matrix. TWEAK, TNF‐like weak inducer of apoptosis

We previously found that both TWEAK and Fn14 are expressed in the intervertebral disc (Table 1 and Figure 2),^10^, ^14^, ^15^ and that their signal activity inhibited the production of cartilage matrix in the intervertebral disc, and further induced the expression of MMP‐3.^10^ Furthermore, although its effect is slower than that of TNF‐α, TWEAK is involved in degeneration of the intervertebral disc by promoting degradation of the cartilage matrix.^14^

**Table 1:** Summary of our findings about the potential role of TWEAK in disc degeneration

Experiments		
Tissue source/methods	Findings	Ref
Human HD/HE	TWEAK and Fn14 were both expressed	
Mouse IVD/HE, RT‐PCR	TWEAK and Fn14 were both expressed	
Mouse IVD/HE	TWEAK suppressed the production of aggrecan and type 2 collagen	
Mouse IVD/RT‐PCR, ELISA	TWEAK induces the expression of MMP‐3	
Mouse IVD/HE	TWEAK caused the cartilage matrix of the IVD to be degraded	

Abbreviations: ELISA, enzyme‐linked immunosorbent assay; HD, herniated discs; HE, histological evaluation; IVD, intervertebral disc; RT‐PCR, reverse transcription‐polymerase chain reaction; TWEAK, TNF‐like weak inducer of apoptosis.

### 1.2 Disc degeneration mechanism induced by TWEAK and Fn14 signaling

To date, many studies have focused on the mechanism by which major inflammatory cytokines such as TNF‐α and IL‐1β cause disc degeneration.^8^, ^15^, ^16^, ^17^ These inflammatory cytokines have many reported roles in acute phase inflammation such as infection, tumor, and trauma.^18^ TWEAK, by contrast, appears to have a role in chronic inflammatory diseases such as rheumatoid arthritis.^19^ There is a wide range of types of disc degeneration, from those triggered by trauma and infection to those progressing chronically with age‐related changes. We found that TWEAK is involved in the early state of disc degeneration and is constitutively expressed in no irritated intervertebral discs, by contrast with TNF‐α, through a chronic inflammation mechanism.^10^, ^14^ Thus, TWEAK is considered involved in the early state of disc degeneration. As such, we believe TWEAK and Fn14 signaling are promising therapeutic targets for the treatment of IVD degeneration.^20^

However, although findings have been obtained in tissue culture of intervertebral discs from mouse and human surgical samples, how TWEAK and Fn14 contribute to intervertebral disc degeneration and the effect of their signal in vivo remains unknown. TWEAK neutralizing antibodies have been administered systemically in a mouse model of rheumatoid arthritis (RA), and as a consequence inflammation in the joints was suppressed, which holds promise for applications in vivo.^11^

### 1.3 Migration of immunocompetent cells by TSLP and its role in disc degeneration

## 2 FAILURE OF IMMUNE TOLERANCE AND TSLP

Thymic stromal lymphopoietin (TSLP) is an IL‐7‐related cytokine that is produced mainly by epithelial cells of trachea and skin, and is involved in regulating the function of immunocompetent cells that cause allergic diseases, such as bronchial asthma and atopic dermatitis.^21^ Its potential as a therapeutic target is being actively pursued.^21^ TSLPs are produced by epithelial cells in response to exogenous stimuli such as allergens and microbial infections,^22^, ^23^ upon which immunocompetent cells such as dendritic cells, mast cells, and eosinophils are activated through activation of TSLP receptors.^24^

TSLP is highly expressed in diseased tissues of patients with allergic diseases such as asthma and atopic dermatitis, and analysis using genetically modified mice revealed that the action of TSLP is required to produce antigen‐specific allergic conditions. It is possible that TSLP produced by epithelial cells when exposed to exogenous stimuli acts on innate immune cells such as dendritic cells (DC), mast cells, eosinophils, and natural killer T (NKT) cells, which have an important role in the early stage of inflamation.^24^, ^25^ By contrast, the role of TSLP and TSLP receptors in bone and cartilage diseases has not been elucidated until recently. TNF‐α stimulation of synovial cells from rheumatoid arthritis patients induces expression of TSLP, and the blood concentration of TSLP in rheumatoid arthritis patients is elevated compared with the concentration in patients with an osteoarthritic knee.^22^, ^23^

Angiogenesis is a key pathophysiology in many inflammatory diseases such as tumors and traumas.^26^, ^27^, ^28^ In our previous work, we have clarified that neovascularization occurs in the process of natural regression of intervertebral disc herniation.^8^ In this process of degeneration, blood vessel invasion from the endplate occurs, and may be a mechanism as well as a result of degeneration.^29^ Immune tolerance of the intervertebral disc, which is an avascular organ, may be considered dysfunctional. We hypothesized that TSLP might also be involved in the early stages of the immune inflammatory response that occurs in this process. Specifically, (a) the expression of TSLP by the cells of the annulus ring may be induced by external stimulation. (b) TLSP expression plays a role in the migration and infiltration of immunocompetent cells into the IVD.

### 2.1 Roles for TSLP in disc degeneration and spontaneous hernia resorption suggested by our research

**Figure 3:**
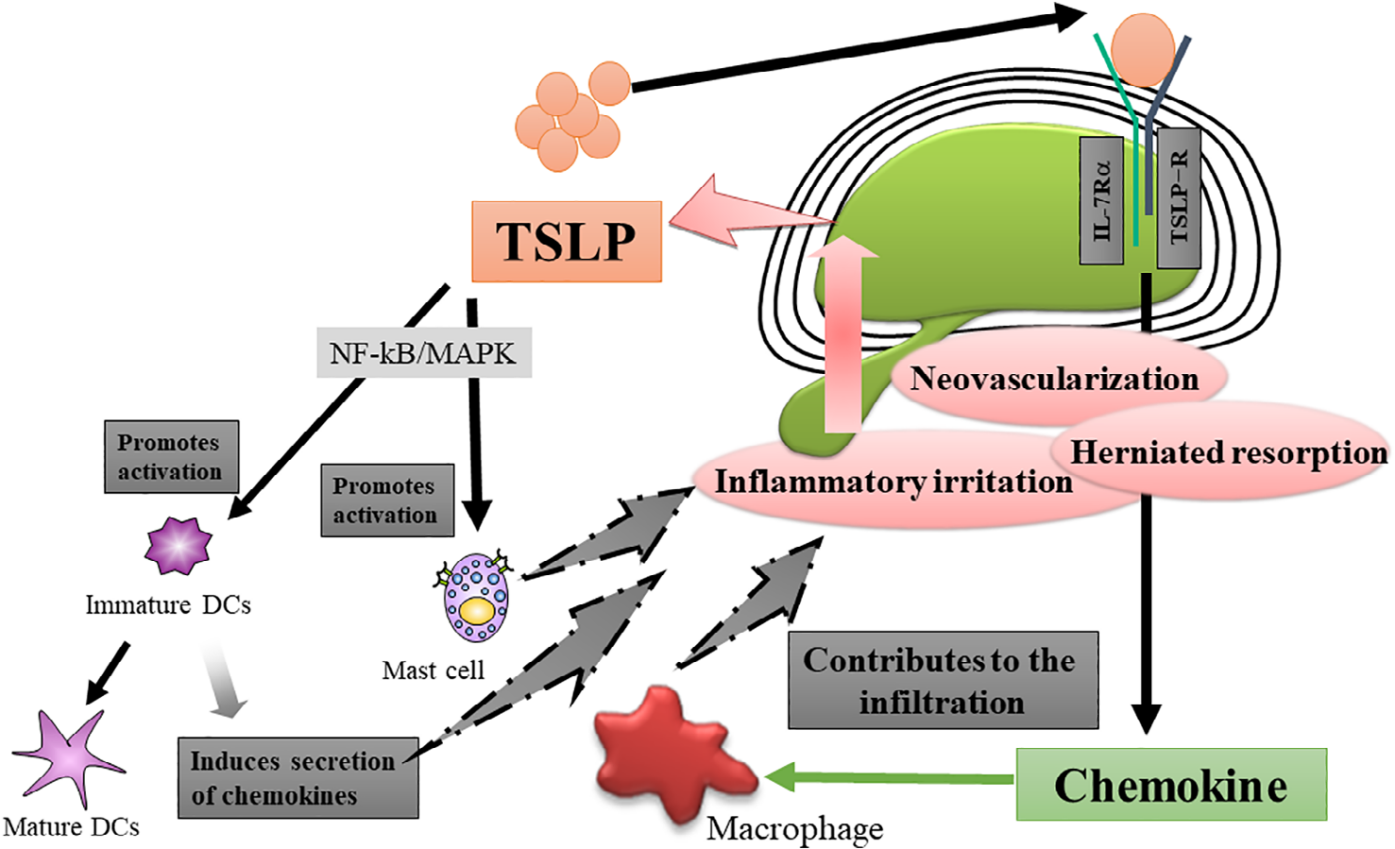
Schematic overview of a proposed mechanism of disc inflammation by TSLP based on our previous studies. TSLP is expressed by the annulus fibrosus when exogenous stimulation applied to the intervertebral disc promotes the production of chemokines by the intervertebral disc. Inward migration of macrophages is involved in the early stages of the inflammation of the intervertebral disc. NF‐κB, nuclear factor‐kappa B; MAPK, mitogen‐activated protein kinase; IL‐7, interleukin‐7; DC, dendritic cells

From our previous work, we propose that TSLP is expressed by the annulus fibrosus when exogenous stimulation applied to the intervertebral disc promotes the production of MCP‐1 and MIP‐1α by the intervertebral disc (Table 2 and Figure 3),^9^, ^30^thereby preventing migration surrounding immunocompetent cells. Inward migration of macrophages is involved in the early stages of the inflammation of the intervertebral disc.^9^ Additionally, endogenous TGF‐β activity limits TSLP expression in intervertebral disc tissue in the steady states by suppressing NF‐κB activation.^30^

**Table 2:** Summary of our findings about the potential role of TSLP in disc degeneration and spontaneous hernia resorption

Experiments		
Tissue source/methods	Findings	Ref
Human HD/HE	TSLP and TSLPR are both expressed	
Mouse IVD/HE, RT‐PCR	TSLP and TSLPR are both expressed	
Mouse IVD/RT‐PCR, ELISA	TSLP was expressed stimulated with TNF‐α, IL‐1β, or LPS	
Mouse IVD/RT‐PCR, ELISA	TSLP induces expression of M‐CSF, MCP‐1, and MIP‐1α	
Mouse IVD/migration assay	TSLP promotes migration of macrophages	
Mouse IVD/HE, RT‐PCR	TGF‐β is constantly expressed by IVD and regulates their expression of TSLP	

Abbreviations: ELISA, enzyme‐linked immunosorbent assay; HD, herniated discs; HE, histological evaluation; IL‐1β, interleukin‐1β; IVD, intervertebral disc; LPS, lipopolysaccharide; M‐CSF, macrophage colony stimulating factor; MCP‐1, monocyte chemoattractant protein‐1; MIP‐1α, macrophage inflammatory protein 1 alpha; RT‐PCR, reverse transcription‐polymerase chain reaction; TGF‐β, transforming growth factor‐β; TNF‐α, tumor necrosis factor‐α; TSLP, thymic stromal lymphopoietin.

### 2.2 Impact of the research so far and future prospects

TSLP is highly expressed in diseased tissues of patients with allergic diseases such as asthma and atopic dermatitis, and analysis using genetically modified mice revealed that the action of TSLP is required to produce antigen‐specific allergic conditions.^24^ It is possible that TSLP produced by epithelial cells when exposed to exogenous stimuli acts on innate immune cells such as dendritic cells (DC), mast cells, eosinophils, and natural killer T (NKT) cells, which have an important role in the early stage of inflamation.^24^, ^25^ By contrast, the role of TSLP and TSLP receptors in bone and cartilage diseases has not been elucidated until now. TNF‐α stimulation of synovial cells from rheumatoid arthritis patients induces expression of TSLP, and the blood concentration of TSLP in rheumatoid arthritis patients is elevated compared with the concentration in patients with an osteoarthritic knee.^22^, ^23^ In a subsequent study,^9^ we found that TSLP was highly expressed in surgical samples of patients with disc herniation, and that the supernatants of TSLP‐activated mouse intervertebral disc cultures had the capacity to induce macrophage migration in an MCP‐1‐dependent manner in vitro.^9^ It will be of great importance in the field of orthopedics to elucidate how TSLP is involved in inflammatory mechanisms in osteochondral tissue.

Further study in vivo and analyzing the presence of TWEAK and TSLP in healthy human tissues should be carried out to support our data obtained in vitro.

## 3 CONCLUSION

Taken together, our previous work illustrates the ability of inflammatory cytokines to induce intervertebral disc degeneration and spontaneous hernia resorption by various mechanisms.

## CONFLICT OF INTEREST

The authors declare no potential conflict of interest.

## AUTHOR CONTRIBUTIONS

T.O. analyzed and interpreted the data was a major contributor in writing the manuscript. H.H. was editorial supervisor of this study. Both authors read and approved the final manuscript.
